# Antitumoral effects of γCdcPLI, a PLA_2_ inhibitor from *Crotalus durissus collilineatus* via PI3K/Akt pathway on MDA-MB-231 breast cancer cell

**DOI:** 10.1038/s41598-017-07082-2

**Published:** 2017-08-01

**Authors:** Sarah N. C. Gimenes, Daiana S. Lopes, Patrícia T. Alves, Fernanda V. P. V. Azevedo, Lara Vecchi, Luiz R. Goulart, Thais C. S. Rodrigues, André L. Q. Santos, Vera L. de C. Brites, Thaise L. Teixeira, Cláudio V. da Silva, Matheus H. Dias, Samuel C. Teixeira, Renata S. Rodrigues, Kelly A. G. Yoneyama, Ricardo A. Oliveira, Veridiana de M. Rodrigues

**Affiliations:** 1 Federal University of Uberlandia, Uberlandia, MG Brazil; 2 Butantan Institute, São Paulo, Brazil

## Abstract

Phospholipases A_2_ **(**PLA_2_s) overexpression is closely associated with the malignant potential of breast cancers. Here, we showed for the first the antitumoral effects of γCdcPLI, a PLA_2_ inhibitor from *Crotalus durissus collilineatus* via PI3K/Akt pathway on MDA-MB-231 cell. Firstly, γCdcPLI was more cytotoxic to MDA-MB-231 breast cancer cells than other cell lines (MCF-7, HeLa, PC3 and A549) and did not affect the viability of non-tumorigenic breast cell (MCF 10A). In addition, γCdcPLI induced modulation of important mediators of apoptosis pathways such as p53, MAPK-ERK, BIRC5 and MDM2. γCdcPLI decreased MDA-MB-231 adhesion, migration and invasion. Interestingly, the γCdcPLI also inhibited the adhesion and migration of endothelial cells and blocked angiogenesis by inhibiting tube formation by HUVECs *in vitro* and sprouting elongation on aortic ring assay *ex vivo*. Furthermore, γCdcPLI reduced the production of vascular endothelial growth factor (VEGF). γCdcPLI was also able to decrease PGE2 levels in MDA-MB-231 and inhibited gene and protein expression of the PI3K/Akt pathway. In conclusion, γCdcPLI showed *in vitro* antitumoral, antimestatatic and anti-angiogenic potential effects and could be an attractive approach for futures studies in cancer therapy.

## Introduction

Breast cancer is the second most common cancer in women while new cases worldwide are increasing every year. According to the National Center for Health Statistics, in the U.S.A. alone, 249,260 new cancer cases and 40,890 deaths were projected for 2016^1^. This disease affects women in developed and developing nations; however, the mortality is highest in low- to middle-income countries^2^, a scenario that illustrates the importance of breast cancer research and new drugs that may control metastatic tumors.

During the past ten years several studies have shown the molecular aspects of breast cancer as being related to loss of cellular contact inhibition, insensitivity to antigrowth signals and resistance to apoptosis^1, 3–5^. Many of these mechanisms involved in breast cancer cell survival are associated with the expression and activity of secretory phospholipases A_2_ (sPLA_2_) and membrane-associated PLA_2_ (M-PLA_2_)^5–12^.

PLA_2_s can hydrolyze membrane phospholipids and release lysophospholipids and free fatty acids, such as arachidonic acid (AA)^11^. AA generates eicosanoids (prostaglandin, leukotriene and thromboxane) which not only are involved in cell proliferation, survival, differentiation, angiogenesis, inflammation and immunity, but also may contribute to the critical steps in cancer growth and metastasis^13, 14^. In addition, PLA_2_s act on cancer cells, through binding on a PLA_2_ receptor, present in the cellular membrane and could stimulate the activation of survival pathway, such as MAPK kinase and PI3K/Akt pathway. Thus, PLA_2_s participate in anti-apoptotic pathways and can be found overexpressed in different types of breast cancer cells; furthermore, their overexpression is closely associated with the malignant potential of breast cancers^6, 15–18^.

Many chemical or natural inhibitors of the PLA_2_ pathway show antitumor effects and may be potential anti-cancer drugs^19–24^. Some non-steroidal anti-inflammatory drugs that inhibit the prostaglandin pathway (COX-2), such as Ibuprofen, have been described as potentially reducing the risk of cancer^24, 25^. Isoliquiritigenin, a flavonoid from *Glycyrrhiza glabra*, induced apoptosis in a human breast cancer cell line (MDA-MB-231) by down-regulating multiple key enzymes in the AA metabolic network, such as sPLA_2_ and deactivation of the PI3K/Akt pathway^21–23, 26^. Donnini *et al*.^20^ also showed cytotoxic and antiproliferative effects on different cancer cells lines, as well as reduction of tumor growth in nude mice transplanted with A431 tumor cells treated with a PLA_2_ inhibitor from *Pyton sebae* snake serum. These works open up new pathways to exploring the therapeutic potential of PLA_2_ inhibitors from snake serum.

Recently, we isolated γCdcPLI, a PLA_2_ inhibitor from *Crotalus durissus collilineatus* (*C. d. collilineatus*) snake serum^27^. The glycoprotein γCdcPLI of 22,344 Da is composed of α-helices (22%) and β-sheets (29%) and is capable of forming oligomers with different numbers of monomers according to temperature. The γCdcPLI inhibits the enzymatic, cytotoxic and myotoxic activities of many PLA_2_s, including the cytotoxic effect on endothelial cells (tEnd) induced by BnSP-7, a PLA_2_ from *Bothrops pauloensis* snake venom.

Here we showed for the first time, the antitumoral, antimetastatic and anti-angiogenic effects of γ-type PLA_2_ inhibitor from snake serum on breast cancer cell via modulation of the PI3K/Akt pathway. The γCdcPLI was cytotoxic to MDA-MB-231 cancer cells and induced modulation of important mediators of apoptosis pathways. Additionally, we showed that γCdcPLI was capable of decreasing MDA-MB-231 adhesion, migration and invasion, and also inhibited the adhesion and migration of endothelial cells (HUVEC). The γCdcPLI also blocked angiogenesis by inhibiting tube formation by HUVECs and significantly reduced the production of vascular endothelial growth factor (VEGF). Moreover, γCdcPLI also inhibit the sprouting elongation on aortic ring assay *ex vivo*. Finally, to elucidate the action mechanism of γCdcPLI, we investigated its involvement in the PLA_2_ pathway, and γCdcPLI was able to decrease PGE2 levels in MDA-MB-231 cells, inhibited gene and protein expression of the PI3K/Akt pathway.

## Results

### γCdcPLI induces cytotoxicity in cancer cell lines

**Figure 1:**
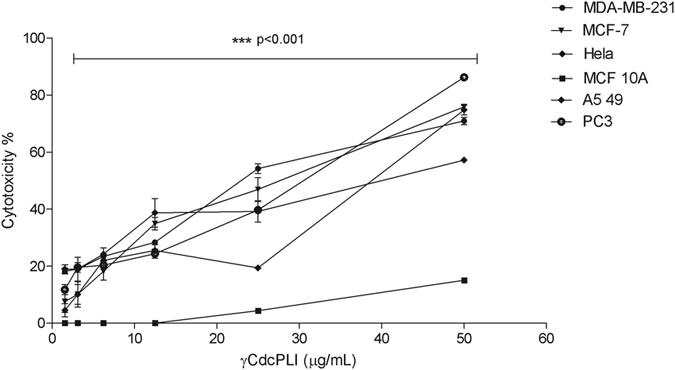
Cytotoxicity induced by γCdcPLI in breast cells by MTT cytotoxicity assay. Breast cancer cells (MDA-MB-231 and MCF-7), Lung cancer cell (A549), Prostate cancer cell (PC3), Cervix cancer cell (HeLa) and the non-tumorigenic breast cells (MCF 10A) were treated with γCdcPLI (50, 25, 12.5, 6.25, 3.125 or 1.56 µg/mL) for 24 h. All data are expressed as mean ± S.E.M and procedures were carried out in triplicate; statistically significant, ***p < 0.001, treatments compared to control (MCF 10A).

Firstly, we investigated the effects of γCdcPLI on the viability of cancer cells using the MTT assay. As shown in Fig. 1 and Table 1 γCdcPLI inhibited growth of cancer cells in a concentration-dependent manner with IC_50_ value of 41 μg/ml for A549 cells (Human Lung Cancer cells), 38 μg/ml for HeLa cell (Human Cervix Cancer cells), 30 μg/ml for PC3 cells (Human Prostate Cancer cells), 28 and 25 μg/ml for MCF-7 cells and in MDA-MB-231 cells (Human Breast Cancer cells), respectively. In contrast, in the same concentration of IC_50_ value of MDA-MB-231, γCdcPLI did not significantly affect the viability of non-tumorigenic MCF 10A cells (***p < 0.001). These results suggest that γCdcPLI is more cytotoxic to breast cancer cells (MDA-MB-231 and MCF-7) than others cancer cell lines and to non-tumorigenic breast cells. The triple-negative and highly metastatic breast cancer cells, MDA-MB-231, were subject of γCdcPLI inhibition as showed in the next experiments.

**Table 1:** IC_50_ value of γCdcPLI in different cell cancer line.

Cell Line	IC_50_ value (μg/mL)	Statistic (IC_50_)^a^
MDA-MB-231	25 ± 1.72	—
MCF-7	28 ± 4.1	ns
PC3	30 ± 0.56	***
HeLa	38 ± 3.4	***
A549	41 ± 3.72	***

^a^Compared with IC_50_ value of MDA-MB-231 (***p < 0.001).

### Cell death induced by γCdcPLI in MDA-MB-231 cells

**Figure 2:**
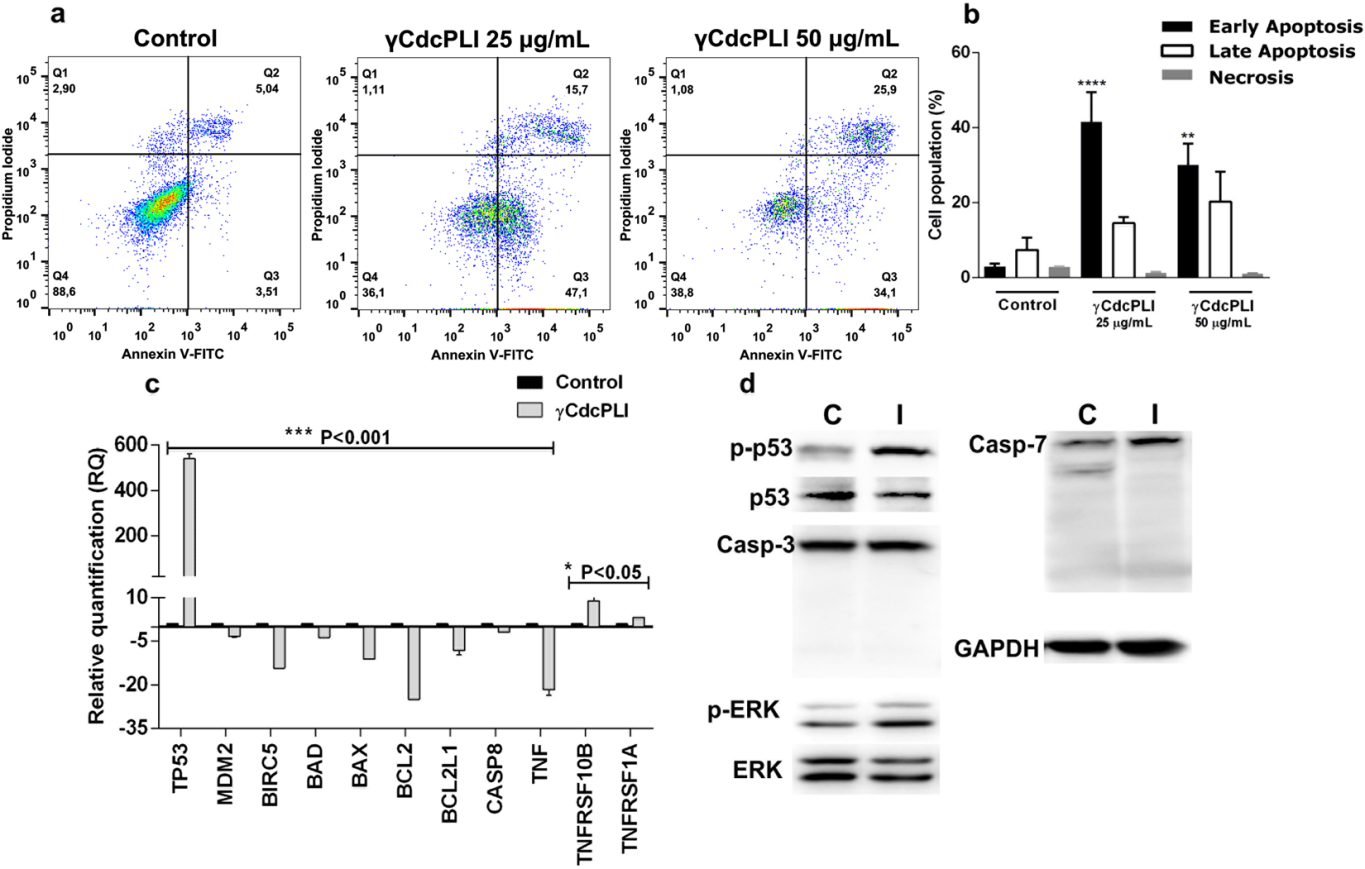
MDA-MB-231 cell death induced by γCdcPLI. (**a**) Analysis of apoptosis by flow cytometry: The representative dotplot acquisitions revealed apoptotic cells and necrotic areas. (**b**) Analysis of apoptosis by flow cytometry: Bar diagram showed percentage of cells in necrosis, early and late apoptosis. (**c**) Relative quantification of apoptosis pathway genes by real-time PCR (TP53, MDM2, BIRC5, BAD, BAX, BCL2, BCL2L1, TNF, TNFRSF10B, TNFRSF1A, CASP8) in MDA-MB-231 cells 24 h after γCdcPLI treatment (25 µg/ml). (**d)** Protein expression of many apoptosis-related proteins by Western Blotting (p-Akt, Akt, P-ERK, ERK and the constitutive protein HPRT and GAPDH) in MDA-MB-231 cells treated with γCdcPLI 25 μg/mL for 24 h. All data are expressed as mean ± S.E.M and all experiments were carried out in triplicate; differences between treatments and controls were analyzed by One-way, Two-way ANOVA and Unpaired t-test. Statistically significant, ***p < 0.001 and **p < 0.005, treatment compared to control.

Next, we investigated whether the cytotoxicity induced by γCdcPLI in MDA-MB-231 cells could be mediated by apoptosis. The results performed by flow cytometry, using annexin V-FITC/PI staining, showed that γCdcPLI treatment was capable of inducing early apoptosis in 47.1% and 34.1% of cells in the respective treatments, 25 µg/mL and 50 µg/mL, when compared to control cells (***p < 0.001 and **p < 0.005); and additionally, the late apoptosis was 15.7% and 25.9% after the treatments (Fig. 2a and b). In addition, the necrosis levels were lower in γCdcPLI-treated cells when compare with the control group, 1.11% and 1.08%. These results showed that apoptosis was more expressive than necrosis (Fig. 2b).

To measure the relative mRNA level of apoptosis-related genes, quantitative real-time analysis was performed (Fig. 2c). Analysis by qPCR-RT utilizing the Human Cancer Pathway Primer Library showed that γCdcPLI (25 µg/mL) was capable of modulating the expression of genes involved in the signaling apoptosis pathway. The γCdcPLI-treatment modulated the expression of genes involved in the intrinsic apoptosis pathways BAD, BAX, BCL2 and BCL2L1, and the extrinsic apoptosis pathways TNF, TNFRS10B, TNFRSF1A and CASP8. Two genes involved in the extrinsic apoptosis pathway were up-regulated, whereas the TNF receptors (TNFRS10B and TNFRSF1A) (**p < 0.05) and all the others were down-regulated (***p < 0.001) (Fig. 2c).

Furthermore, we analyzed genes of p53, another important pathway to apoptosis activation. MDM2 and BIRC5 (Survivin) were down-regulated while in contrast, TP53 was the most up-regulated gene, at a rate nearly 600 times higher than the control cells (***p < 0.001) (Fig. 2c). To confirm the relative mRNA level, we performed the western blotting analysis of p53-activated (p-p53) protein. Our results showed that the p53 level was markedly increased in the MDA-MB-231 malignant cells treated with the PLA_2_ inhibitor (Fig. 2d). In addition, we evaluated the active ERK (p-ERK) levels after γCdcPLI treatment by the western blotting technique. Our results showed that γCdcPLI, increased the active ERK (p-ERK) levels when compared to control, suggesting that can acts in the MAPK-ERK pathway.

Furthermore, we investigated the involvement of caspases 3 and 7 in the apoptosis triggered by γCdcPLI. Caspase 3 or 7 cleavages (activation) were not triggered by the inhibitor. The results showed that cleavages of neither Caspase 3 nor Caspase 7 were affected by the γCdcPLI treatment.

### Anti-metastatic effect in MDA-MB-231

**Figure 3:**
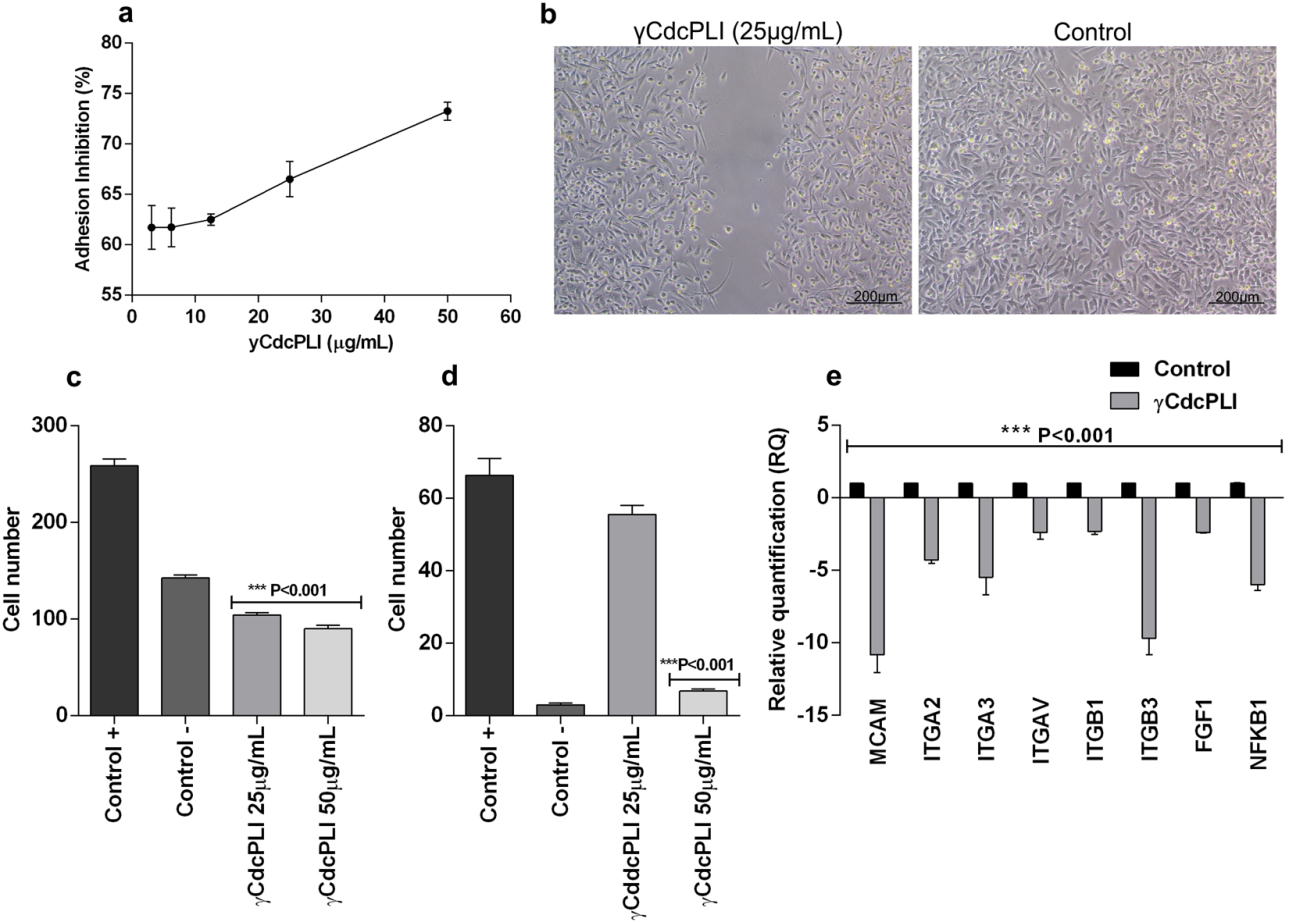
Anti-metastatic effect of γCdcPLI inhibitor. (**a**) Inhibition of adhesion cell by γCdcPLI at different concentrations (50, 25, 12.5, 6.25, 3.125 μg/mL). (**b**) Migration analysis by Wound Healing Assay. The representative images of the wound in MDA-MB231 at 24 h after γCdcPLI treatment (25 μg/mL). (**c**) Inhibition of migration cells through transwell, positive control, negative control and γCdcPLI at 25 and 50 μg/mL. (**d**) Inhibition of invasion cells by Matrigel-transwell assay, positive control, negative control and γCdcPLI at 25 and 50 μg/mL. (**e**) Relative quantification of metastatic genes in MDA-MB-231 cells treated with γCdcPLI at 25 μg/mL. All data are expressed as mean ± S.E.M and all assays were carried out in triplicate; differences between treatments and controls were analyzed by Unpaired t-test. Statistically significant values are represented by ***p < 0.001.

To evaluate the γCdcPLI-induced anti-metastatic effect on the MDA-MB-231 cells, we investigated the cellular adhesion, migration and invasion, three important events in the metastatic process. The γCdcPLI inhibited cell adhesion in a dose-dependent manner by approximately 60% and 75% at 3.125 µg/mL and 50 µg/mL, respectively (Fig. 3a). In addition, the inhibitor at 25 µg/mL was able to inhibit the cell migration in the wound healing assay, when compared to the control (medium) for 24 hours (Fig. 3b). This result was confirmed by the transwell migration assay, which demonstrated the ability of γCdcPLI (25 and 50 µg/mL) to reduce MDA-MB-231 migration through the transwell (Fig. 3c and Supplementary Fig. 1a) by approximately 60% when compared to positive control after γCdcPLI (25 and 50 µg/mL) treatment.

The effects of γCdcPLI on invasiveness of MDA-MB-231 reduced the invasion capacity of tumor cells in the Matrigel-transwell assay by 87% when compared to the positive control (Fig. 3d and Supplementary Fig. 1b).

Moreover, we analyzed expression of some important genes related to adhesion and proliferation and that participate in a metastatic mechanism, such as MCAM, ITGA2, ITGA3, ITGA4, ITGAV, ITGB1, ITGB3, FGF1 and NFKB1. In these data the γCdcPLI down-regulated many genes involved in cellular adhesion, such as genes that encode integrin (α2, α3, αV, β1 and β3), however did not act on recognition of integrins on cell surface of MDA-MB-231 (Fig. 3e and Supplementary Fig. 3a). Moreover, the inhibitor treatment decreased the expression of genes that encode adhesion molecules, such as MCAM, which encode the Cell Surface Glycoprotein MUC18; and growth factor, as a member of Fibroblast Growth Factor encoded by FGF1 gene, both molecules key to the cell adhesion and tumor progression. In addition, the γCdcPLI treatment decreased the expression of the NFKB1 gene, which encodes the NFκβ factor, also important to the survival and metastasis of cancer cells.

### Cytotoxicity effect in HUVEC cells

**Figure 4:**
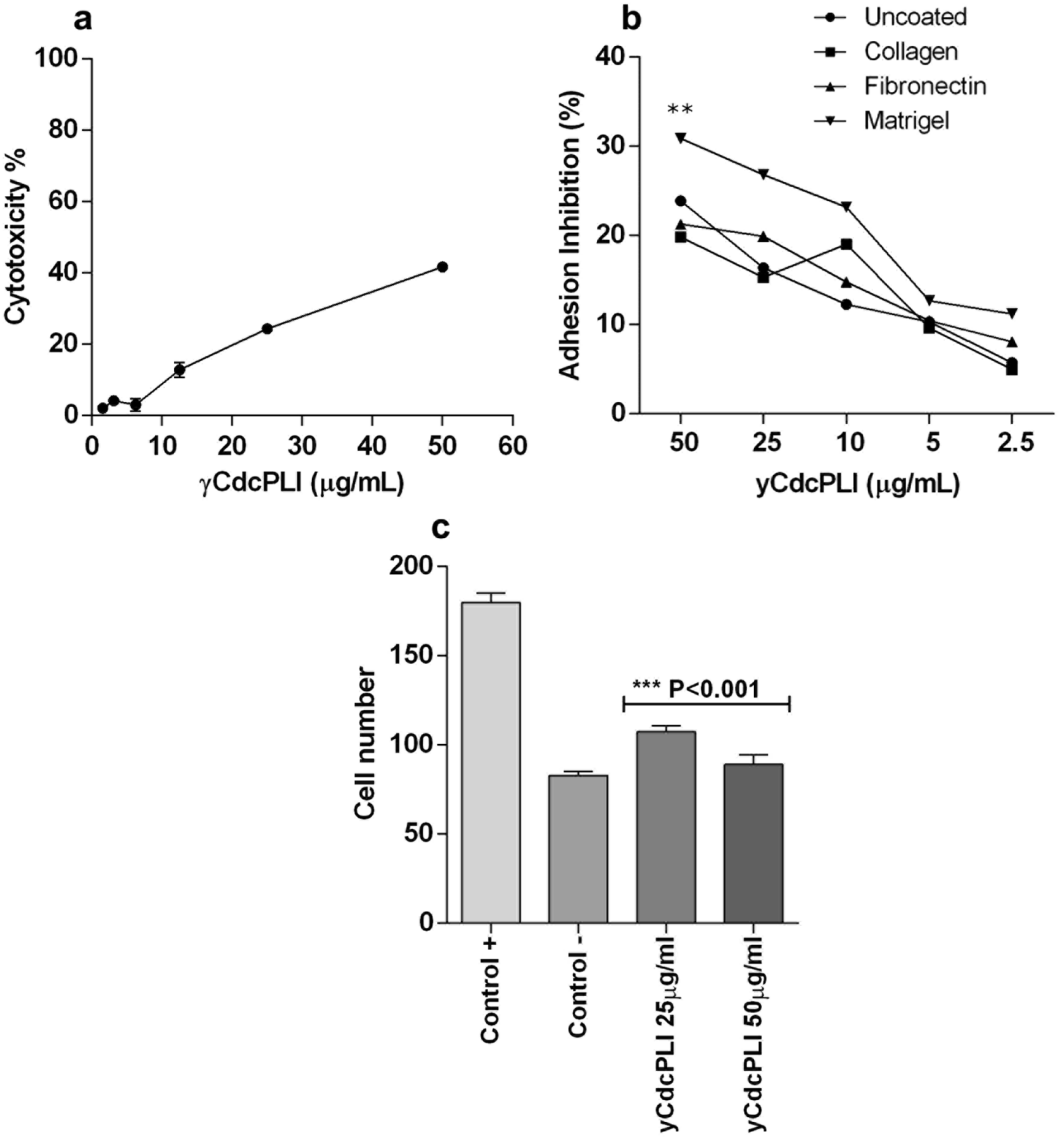
Cytotoxicity induced by γCdcPLI in HUVEC cells and effect of γCdcPLI inhibitor on adhesion and migration of HUVECs cells. (**a**) Cytotoxicity percentage of HUVECs treated with γCdcPLI (50, 25, 12.5, 6.25, 3.125, 1.560 µg/mL) for 24 h. (**b**) Adhesion cell inhibition by γCdcPLI at different concentrations (50, 25, 10, 5 and 2.5 μg/mL). (**c**) Migration analysis by Transwell assay, positive control, negative control and γCdcPLI at 25 and 50 μg/mL. All data are expressed as mean ± S.E.M and all assays were carried out in triplicate; differences between different extracellular matrix (ECM) components, treatments and controls were analyzed by One-way ANOVA and Unpaired t-test. Statistically significant (**p < 0.05; ***p < 0.001).

The γCdcPLI at different concentrations (50, 25, 12.5, 6.25, 3.125 and 1.560 µg/mL) induced cytotoxicity in a concentration-dependent manner in HUVEC cells. Nevertheless, γCdcPLI showed lower cytotoxicity in HUVEC cells. In this case the IC_50_ value of γCdcPLI in HUVEC was not determined and the major concentration (50 µg/mL) induced a cytotoxicity of just 40% (Fig. 4a).

### Anti-angiogenic effect of γCdcPLI in HUVEC cells

#### Adhesion, migration

To evaluate the anti-angiogenic effect induced by γCdcPLI, we investigated the adhesion and migration of HUVECs. The data show that the inhibitor was able to reduce the cell adhesion in a concentration-dependent manner in the different extracellular matrix (ECM) components evaluated, namely collagen, fibronectin and matrigel (Fig. 4b). The γCdcPLI at 50 μg/mL inhibited 34, 20 and 23% of HUVEC adhesion in matrigel, collagen and fibronectin, respectively. These data showed that the inhibitor treatment was more efficient in inhibiting the adhesion of HUVEC cells in Matrigel than other extracellular matrix proteins.

Furthermore, γCdcPLI reduced the migration capacity of HUVEC cells (Fig. 4c and Supplementary Fig. 2), via transwell assay showed a lower number of cell migrated after inhibitor treatment in comparison with the positive control (***p < 0.001), which corresponds to 34 and 45% of migration at 25 and 50 µg/mL, respectively.

#### Gene expression and Integrin measurement in HUVEC cells

**Figure 5:**
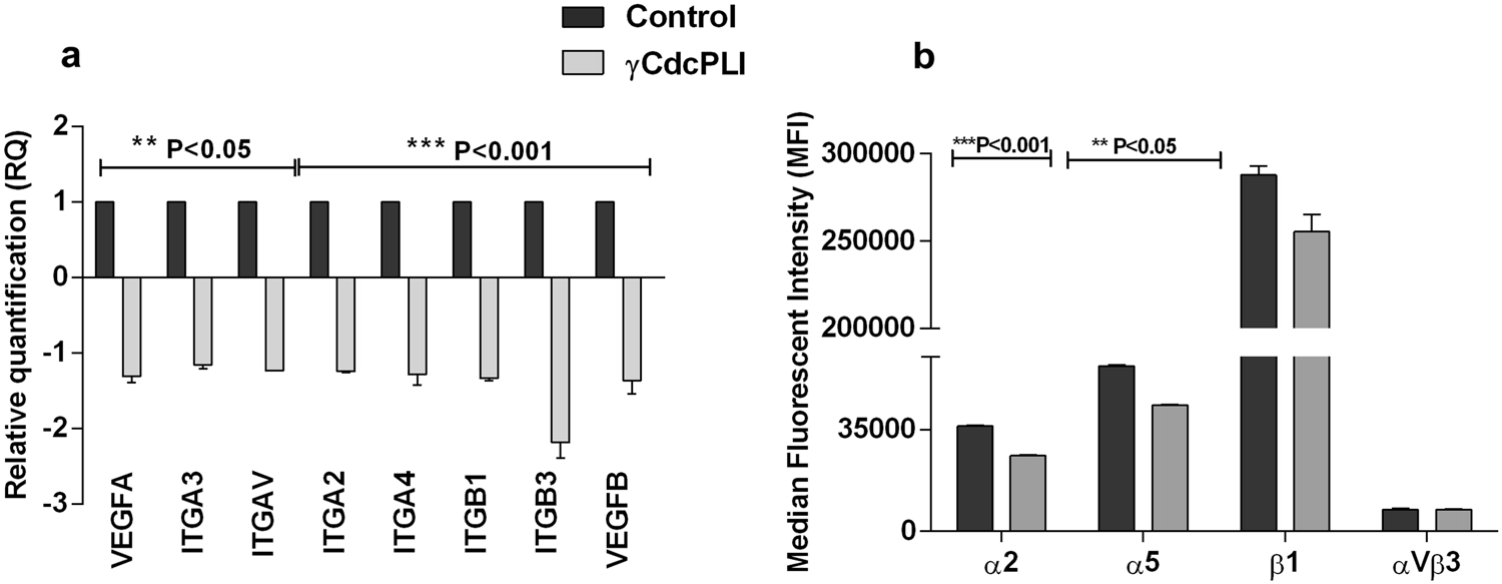
Gene and Integrin expression in HUVEC cells. (**a**) Relative quantification of genes expressed in HUVEC cells (VEGFA, VEGFB, ITGA2, TIGA3, ITGA4, ITGB1, ITGB3 and ITGBV) treated with γCdcPLI 25 μg/mL. (**b**) The graphic representation of median values of integrin expression in HUVEC cells treated with γCdcPLI 50 μg/mL. All data are expressed as mean ± S.E.M and all experiments were carried out in triplicate; differences between treatments and controls were analyzed by Unpaired t-test. Statistically significant: **p < 0.05 and ***p < 0.001.

To evaluate the γCdcPLI modulation of gene expression involved with angiogenesis we analyzed the relative mRNA level of the growth factor (VEGFA and VEGFB) and integrin genes (ITGA2, TIGA3, ITGA4, ITGB1, ITGB3 and ITGBV). As shown in Fig. 5a, the inhibitor treatment at 25 μg/mL was capable of down-regulating these angiogenic genes, in comparison with control cells.

In order to support these findings, we evaluated some integrins by flow cytometry. The data show that γCdcPLI at 50 μg/mL was able to reduce the recognition of α2 and α5 integrins on the cell surface of HUVECs. However, HUVEC cells when pre-incubated with γCdcPLI did not affect the levels of the integrins αVβ3 or β1 (Fig. 5b and Supplementary Fig. 3b).

#### Angiogenesis *in vitro* and *ex vivo* assay

**Figure 6:**
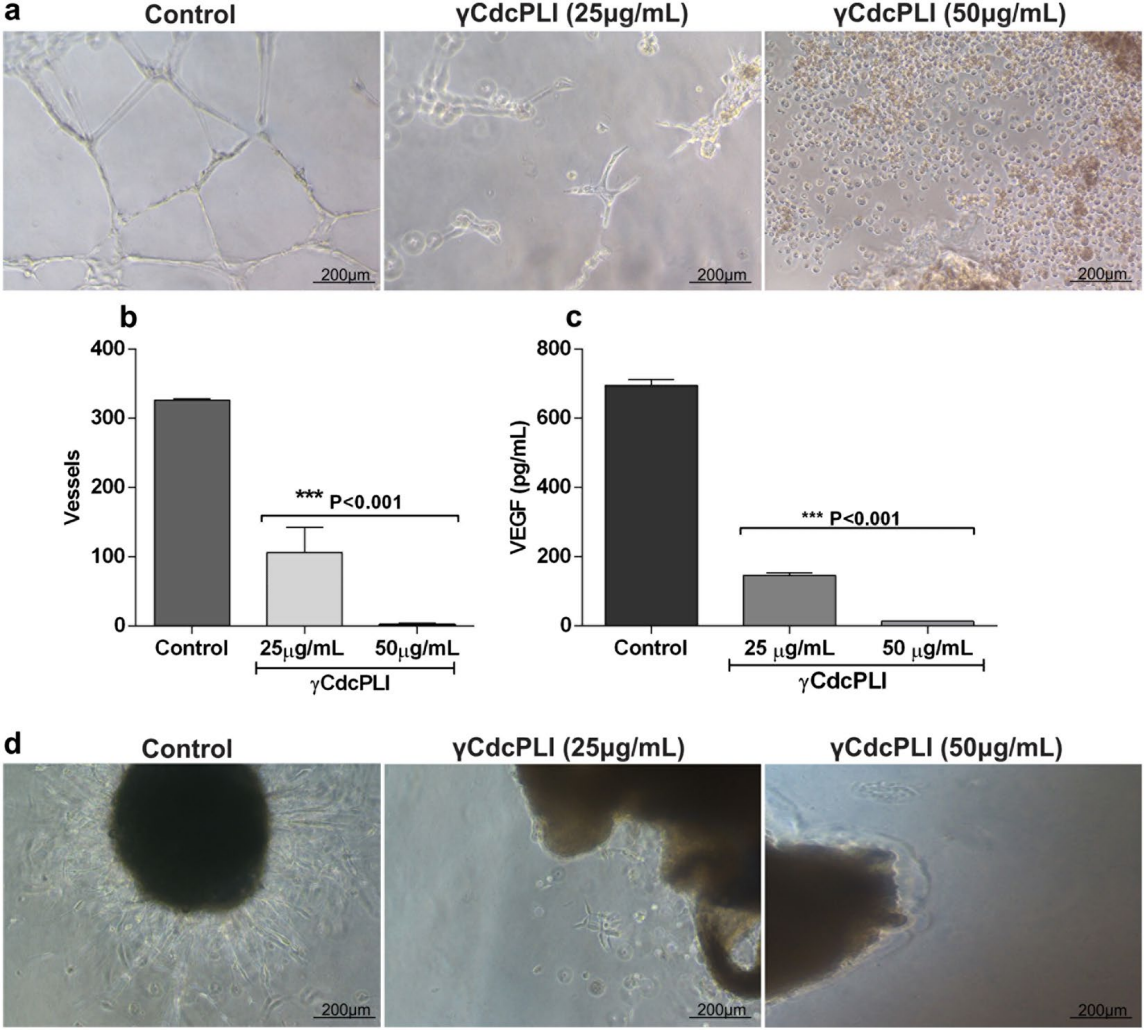
Analysis of *in vitro* and *ex vivo* angiogenesis assay. (**a)** Vessel formation of HUVEC cells when treated with RPMI medium and γCdcPLI at 25 μg/mL for 18 hours. (**b)** Representative quantification of number of vessels formed (**c)** VEGF quantification in HUVEC cell supernatants. (**d)** Sprouting of elongated vessels from the *ex vivo* aortic ring model when treated with medium (control) and γCdcPLI at 25 and 50 μg/mL for 7 days. All data are expressed as mean ± S.E.M and all experiments were carried out in triplicate; differences between treatments and controls were analyzed by Unpaired t-test. Statistically significant: **p < 0.05 and ***p < 0.001.

To analyze the anti-angiogenic effect of γCdcPLI, we first evaluated the vessel formation by HUVEC cells *in vitro* on Matrigel. The γCdcPLI (25 and 50 μg/mL) inhibits the vessels induced by bFGF when compared to the control treatment. Approximately 220 vessels were counted in the control group while the HUVEC cells treated with 25 and 50 μg/mL presented respective decreases in the number of vessels to 105 and 5 (***p < 0.001) (Fig. 6a and b).

In addition, we performed the quantification of vessel growth factor (VEGF) present on HUVEC supernatants from the *in vitro* angiogenesis assay. These data showed that the VEGF released in the untreated HUVEC supernatants was approximately 720 pg/mL versus respective diminutions in the 25 and 50 μg/mL inhibitor treatments of 160 and 10 pg/mL (Fig. 6c) (***p < 0.001).

Furthermore, the *ex vivo* angiogenesis was analyzed in the aortic ring *ex vivo* model. As shown in Fig. 6d the inhibitor treatment at 25 and 50 μg/mL was able to reduce the sprouting of elongated vessels in comparison with the control. To enhance this γCdcPLI property we used the aortic ring *ex vivo* model. The data revealed that inhibitor treatment at 25 and 50 μg/mL for 7 days reduced the number of sprouting elongated vessels in comparison with controls.

### γCdcPLI effects on PLA_2_ pathway in MDA-MB-231

#### Prostanoid levels in MDA-MB-231 supernatants and Modulation of PI3K/Akt pathway

**Figure 7:**
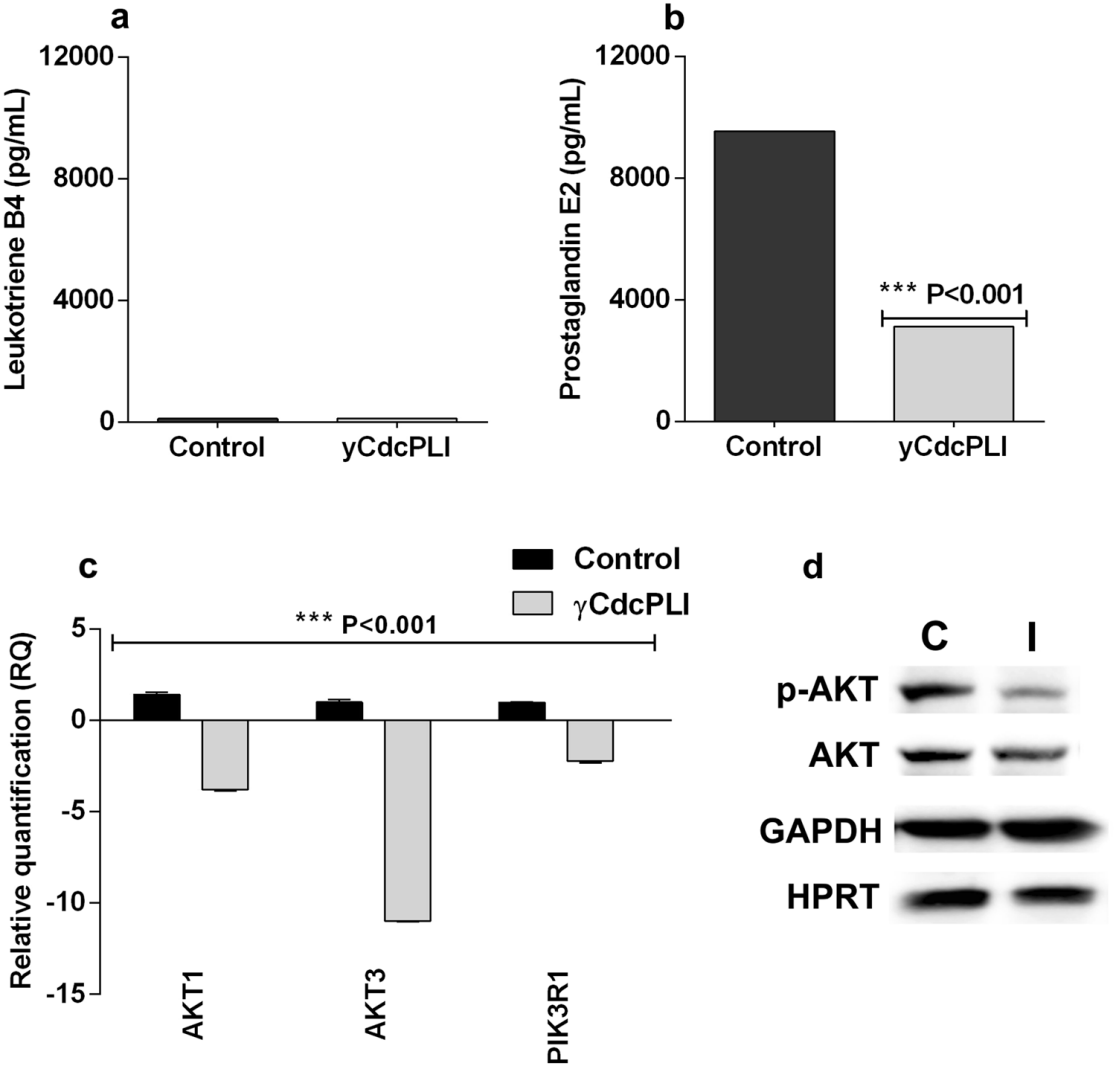
Prostanoid levels in MDA-MB-231 supernatants and modulation of PI3K/Akt pathway. (**a)** Level measurements of prostanoids produced in MDA-MB-231. Leukotriene B4 and Prostaglandin E2 levels in MDA-MB-231 supernatant after treatment with γCdcPLI at 25 µg/mL. (**b)** Relative quantification of genes and protein expressed in MDA-MB-231 cells treated with γCdcPLI 25 μg/mL (Akt1, Akt3 and PIK3R1). (**c**) Protein expression of p-Akt, Akt and the constitutive protein HPRT and GAPDH was used as housekeeping. All assays were carried out in triplicate; differences between treatments and controls were analyzed by Unpaired t-test. Statistically significant: ***p < 0.001.

In order to evaluate the involvement of the PLA_2_ pathway in γCdcPLI effects, firstly we measured the level of prostanoids in MDA-MB-231 supernatants. As shown in Fig. 7a, the leukotriene level in MDA-MB-231 cell supernatant was not significantly affected by γCdcPLI treatment when compared to the control. However, the PGE2 level in supernatant of non-treated cells (control) was 9,556.20 pg/mL versus 3,125.24 pg/mL in γCdcPLI-treated cells (***p < 0.001). Thus, γCdcPLI inhibited PGE2 secretion in MDA-MB-231 cells.

Next, we evaluated the expression of some genes related to PI3K/Akt pathways involved in the maintenance of cell survival and escape from apoptosis. When treated with γCdcPLI 25 μg/mL, the MDA-MB-231 cells showed decreased expression of Akt1, Akt3 and PI3KR1 (Fig. 7b). In order to support these findings, we analyzed the activation pattern expression of PI3K/Akt pathway proteins by western blotting. As shown in Fig. 7c, γCdcPLI treatment at 25 μg/mL inhibited the activity of the PI3K/Akt pathway, decreasing the level of the active form of Akt protein (p-Akt).

## Discussion

PLA_2_ has been described as a carcinogenesis mediator^17, 18, 28^; in this context, some studies have reported the antitumor effect of PLA_2_ inhibition^20, 29^. In this work, we demonstrated that γCdcPLI displays a possible preference for a target in breast cancer cells (MDA-MB-231 and MCF-7) when compared to others cancer cell lines evaluated (HeLa, A549 and PC3). Interestingly, cytotoxic activity of γCdcPLI was significantly lower against a non-tumorigenic breast cell line, showing at the same concentrations lower cytotoxicity in MCF 10A cells than that in MDA-MB-231 cells, an important aspect in discovering a potential drug in cancer therapy.

Thus, we addressed the antitumor, antimetastatic and anti-angiogenic (*in vitro* and in the aortic ring model *ex vivo*) effects of γCdcPLI, a PLA_2_ inhibitor from *Crotalus durissus collilineatus* snake serum on breast cancer cells (MDA-MB-231). MDA-MB-231 cells, are the most commonly used *in vitro* model of triple negative breast cancer, characterized by the absence of estrogen receptor, progesterone receptor and human epidermal growth factor receptor 2. These cells are highly metastatic, tumorigenic and resistant to apoptosis^30–32^.

The cytotoxicity induced by γCdcPLI in MDA-MB-231 cells may be associated with cell death by the apoptosis. Apoptotic cells can be characterized as an early apoptotic process, when exposes the phosphatidylserine (PS) on the cell surface to mediate its recognition by phagocytic cells; and the plasma membrane remains intact. Early apoptotic cells can become late apoptotic cells, when the plasma membrane becomes permeated. Late apoptotic cells are also known as secondary necrotic cells^33, 34^. The main stage of cell death observed in cells treated with γCdcPLI was early apoptosis, which is characterized by Annexin V-positive.

Until now, only one previous work demonstrated the antitumor effect of γ-type PLA_2_ inhibitors from snake serum in cancer cells lines. In this work, they showed cytotoxic, antiproliferative and apoptotic effects on breast cancer cells and other lines cells treated with a PLA_2_ inhibitor from *Pyton sebae* snake serum^20^. However, research studies that seek to investigate the action mechanism of PLA_2_ inhibitors are still scarce.

Thus, in order to elucidate the molecular mechanism used by γCdcPLI to activate apoptosis in MDA-MB-231 cells, we examined the expression of apoptosis markers that characterize extrinsic and intrinsic apoptosis pathways. The intrinsic pathway is mediated through a mitochondrial-dependent mechanism and is regulated by anti-apoptotic (Bcl-2, Bcl-xL, Bcl-w and Mcl-1) and pro-apoptotic members (Bax, Bak, Bad, Bid, Bmf and others)^34^.

PLA_2_ inhibitor (γCdcPLI) induced down-regulation of important anti-apoptotic genes associated with the intrinsic pathway, Bcl-2 and BCL2L1. However, treatment with γCdcPLI down-regulated some pro-apoptotic genes analyzed, such as BAD and BAX. In addition, γCdcPLI treatment was not capable of activating and modulating Caspase 3 or Caspase 7, important proteins related to the apoptosis phase denominated the execution pathway, in which the intrinsic and extrinsic pathways finish, i.e., the end of apoptotic pathway^35^.

Extrinsic pathway signaling has been correlated with transmembrane receptor members such as tumor necrosis factor (TNF) receptors; interestingly the γCdcPLI up-regulated the genes TNFRSF10B and TNFRSFIA, which encode TNF receptors^34, 36^. Nevertheless, the inhibitor has provoked a down-regulation in the expressions of TNF and caspase 8 in treated cells compared to the control. Although our data shows that the γCdcPLI has induced regulation of some genes that prevent cellular apoptosis, its main effect on MDA-MB-231 cells was the regulation of pro-apoptotic genes, which corroborates our observations of cellular death.

Furthermore, we investigated the modulation of important mediators in cellular processes of apoptosis, such as MAPK-ERK and the p53 pathway. The gene TP53 encodes the p53 protein, a tumor suppressor^37–39^, while the expression of p53 in cancer cells has been associated with the expression of pro-apoptotic genes^40^, thus preventing an oncogenic state. Disruption of p53 function promotes cell cycle checkpoint defects, cellular immortalization, proliferation, genomic instability and survival of cancer cells^41, 42^. In the current study, the gene most up-regulated by γCdcPLI was TP53; and we also demonstrated that inhibitor treatment increased the levels of the active form of p53 (p-p53). Apoptosis induced in cancer cells by increasing expression of p53 has been related to such substances as Thymoquinone, a bioactive constituent of black seed oil (*Nigella sativa*), and BDMC-A, an analog of Curcumin (a yellow pigment from rhizomes of *Curcuma longa*)^4, 43, 44^. Furthermore, another important gene involved in the p53 pathway analyzed was BIRC5 (Survivin), a member of the inhibitor of apoptosis protein (IAP) family^45^. Overexpression of the BIRC5 gene in breast cancer cells (MCF-7) increases MDM2 levels and decreases p53 gene expression, thus inhibiting the apoptotic effect induced by the p53 pathway^46^. MDM2 is overexpressed in many cancer cell lines and binds on p53, leading to cancer cell escape from p53-regulated control^47^. Thus, γCdcPLI induced down-regulation of MDM2 and BIRC5 in MDA-MB-231 cells, results that may be corroborated with the data indicating overexpression of TP53 and p-p53 and apoptosis observed in the present work.

In addition, our results demonstrate that γCdcPLI also increased active ERK (p-ERK) levels. The MAPK-ERK pathway is important for cell proliferation; however, it can be activated by cellular stress and induce apoptosis^48^. Therefore, this pathway may be also activated to the MDA-MB-231 apoptosis induced by γCdcPLI.

The six hallmarks of cancer include, besides programmed cell death (apoptosis), tissue invasion, metastasis and angiogenesis^49^. The metastatic process of cancer cells is the most important event responsible for tumor malignancy, and is regulated by capacity for cellular adhesion, migration and invasion^3^. In this context, we showed that γCdcPLI was capable of decreasing the MDA-MB-231 adhesion, migration and invasion by wound healing and transwell in *in vitro* models. Furthermore, the inhibitor treatment was able to down-regulate the gene expression of some integrins (α2, α3, αV, β1 and β3), growth factor and adhesion molecules (FGF-1 and MCAM).

The integrins are a family of cell adhesion receptors composed of α and β subunits that form 24 heterodimers and mediate cell adhesion and interaction with the extracellular matrix^50, 51^. The gene that encodes β3 integrin was the most down-regulated gene in the MDA-MB-231 cell when treated with γCdcPLI. The β3 integrin is responsible for recognizing and binding to such extracellular proteins as fibronectin, laminin and vitronectin. In cancer progression, integrins, adhesion molecule and growth factor are important for controlling survival, differentiation and cell proliferation^52–56^.

Fibroblast growth factor (FGF) is another important regulator of the growth, differentiation and angiogenesis process. The binding of FGF to its receptor leads to an intracellular signaling cascade, mainly on the Ras/MAPK pathway. Moreover, the FGF signaling promotes tumor progression^57, 58^. The MCAM is a membrane calcium-independent glycoprotein adhesion molecule and contains several motifs responsible for protein kinase recognition. This recognition suggests that MCAM participates in different signaling pathways in the cell, while some studies show that MCAM is abnormally expressed in different tumors and is associated with the cancer progression and metastasis process, in breast, lung and prostate cancer^59, 60^. Taken together, these data demonstrate that γCdcPLI can modulate a cell adhesion mechanism and consequently interfere in the MDA-MB-231 metastasis process.

Angiogenesis is an important process involved in metastasis and tumor remodeling^61, 62^ and is characterized by forming new vessels from preexisting blood vessels^63, 64^. When the cells receive an angiogenic stimulus, the endothelial cells act to migrate, proliferate, adhere and align to form the new blood vessels^65, 66^. Tumor angiogenesis is important for supplying nutrients to cancer cells, which present increased metabolism and invade adjacent tissues.

The human endothelial cells (HUVECs) when treated with γCdcPLI lose their capacity to adhere to extracellular matrix proteins and migrate at low cytotoxic concentrations (50 μg/mL). Moreover, the inhibitor treatment down-regulated the gene expression of integrins (α2, α3, α4, αV, β1 and β3) whereas an integrin analysis by cytometry flow assay showed that the γCdcPLI treatment decreased the recognition of α2 and α5 integrin in HUVEC cells. These data may be related to a possible interaction of γCdcPLI with integrin receptors. Until now, no work has demonstrated the capacity of PLA_2_ inhibitors to recognize and bind to integrins. However, some toxins from snake venom can bind to integrins and modulate an antiangiogenic effect, such as Lectins and Phospholipases A_2_^67–69^. The α2 and α5 integrins present in the cells are receptors for collagen and fibronectin in extracellular matrix, respectively^70^. Interestingly, our adhesion inhibition data showed the best inhibition values using Matrigel and collagen as substrate. Taken together these results suggest that γCdcPLI was capable of inhibiting HUVEC adhesion by decreasing the expression and/or recognition of some integrins important for cell adhesion in the extracellular matrix.

The γCdcPLI blocked angiogenesis inhibiting tube formation by HUVECs induced by bFGF. One possible means of blocking angiogenesis is by inhibition of pro-angiogenic factors secreted by tumor cells such as VEGF. This growth factor is a major pro-angiogenic protein expressed in 60% of breast cancer patients^71^. It is responsible for stimulating the proliferation and migration of endothelial cells in tumor angiogenesis, thereby providing conditions for the formation of new vessels^72, 73^. Importantly, we found that γCdcPLI significantly reduced the production of VEGF in HUVECs, thus suggesting one possible mechanism through which γCdcPLI inhibits angiogenesis.

In order to corroborate our *in vitro* data, we performed the *ex vivo* aortic ring assay, in which occurs sprouts grow out from the aortic sections. Our results showed that γCdcPLI decreased the capacity of a preexisting vessel to form new sprouting for elongation. Some works identified by this *ex vivo* model, the presence of endothelial and supporting cells (pericytes) through immunofluorescence staining and also showed different aspects of cellular and molecular of angiogenesis^74–76^. According to these researches, this assay could confirm the potential anti-angiogenic of γCdcPLI. Abu, *et al*., (2016) also demonstrated the antitumor, antimetastatic and antiangiogenic properties of a natural compound, Flavokawain B, from the Kava-kava plant (*Piper methysticum*)^77^. In this work the author showed that Flavokawain B, besides its antitumor effect on MCF-7 and MDA-MB-231 cells, regulated the angiogenesis process by diminishing vessel formation in an *in vitro* model using HUVEC cells, and the *ex vivo* model by the aortic ring assay^78, 79^.

The γCdcPLI effects in MDA-MB-231 cells can be related to PI3K/Akt pathway, which is activated by eicosanoids (PGE2 and LTB4)^80, 81^. This pathway has shown an essential role in proliferation, survival, angiogenesis and cancer progression^82^. Therefore, blocking any one of the key pathways in the AA-metabolic network might inhibit progression of breast cancer tumors. The γCdcPLI could be capable of interfering in the AA pathway by decreasing the PGE2 level in MDA-MB-231 cellular supernatant. PGE2 is overexpressed in breast cancer cells, especially in highly invasive and metastatic lines (MDA-MB-231). Moreover, γCdcPLI inhibited gene expression of PI3KR1, Akt1, Akt3 and decreased the active form of Akt (p-Akt). Some compounds demonstrate antitumor properties against breast cancer cells by down-regulating the activity of the PI3K/Akt pathway, while studies have shown that down-regulation of multiple enzymes pertaining to the AA pathway can inhibit cell growth and apoptosis of breast cancer cells^29, 83^.

**Figure 8:**
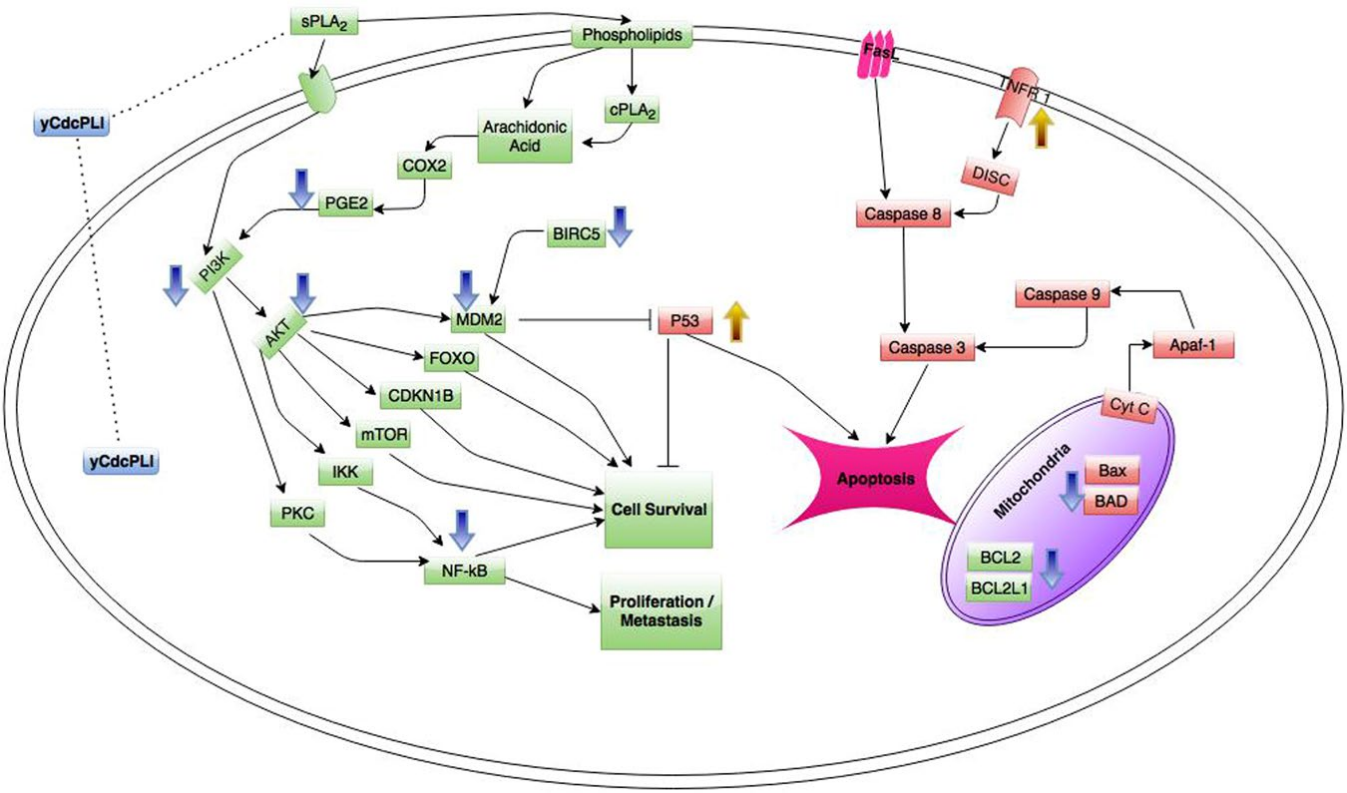
Representative model of some signaling pathways in a human breast cancer cell (MDA-MB-231) that can be modulated by γCdcPLI. The PLA_2_ inhibitor interferes in gene expression of different proteins involved in proliferation and metastasis, cell survival and apoptosis signaling pathways. PLA_2_, phospholipase A_2_; PGE2, prostaglandin E2; COX, cyclooxygenase; PI3K, phosphoinositide-3-kinase; CDKN1B, cyclin-dependent kinase inhibitor 1B; FOXO, forkhead box, sub-group O; MDM2, survivin; Apaf-1, apoptotic peptidase activating factor 1; FasL, Fas ligand; TNFR1, TNF receptor; PKC, protein kinase C; NF-kB, nuclear factor of kappa light; Rec, PLA_2_ receptor; represents up-regulation; represents down-regulation, represents a γCdcPLI suggested target; Blue arrows means down-regulation and Orange arrows means up-regulation.

Finally, the Fig. 8 summarizes the γCdcPLI effects in a human breast cancer cell (MDA-MB-231), showing modulation of gene and protein expression of different molecules involved in proliferation and metastasis, cell survival and apoptosis signaling pathways. The mechanism of PLA_2_ inhibition could initiate different molecular response showed by the current work, such as inhibition of adhesion cells and angiogenesis, activation of apoptotic pathway and modulation of survival pathway (p53, integrins and PI3K/Akt). We propose γCdcPLI might recognize secreted PLA_2_ (sPLA_2_) and/or cytosolic PLA_2_ (cPLA_2_) and suppress the capacity of lipid mediator release, similarly to other synthetic PLA_2_ inhibitors^84^. Nevertheless, the full elucidation of a γCdcPLI mechanism that leads to apoptosis of MDA-MB-231 cells requires further experimental approaches, such as discovering the target of the inhibitor in cancer cells. Thus, the γCdcPLI may be a potential model of anti-cancer drug via modulation of the PI3K pathway, which is associated with growth and survival of cancer cells.

## Material and Methods

### γCdcPLI purification

The PLA_2_ inhibitor γCdcPLI was purified from *C. d. collilineatus* serum by two sequential chromatographic steps, similarly to the methodology described by Gimenes, *et al*.^27^.

### Cell culture

The Human breast cancer cell line (MDA-MB-231 and MCF-7), a non-tumorigenic breast cell line (MCF 10A), Human prostate cancer cell line (PC3), Human Lung cancer cell line (A549), Human cervix cancer cell line (HeLa) and the Human endothelial cells (HUVEC) were obtained from the American Type Culture Collection (ATCC), and maintained at 37 °C in a humidified incubator containing 5% CO_2_. MDA-MB-231 and MCF-7 cells were cultivated in IMDM medium supplemented with 10% fetal bovine serum, 100 U/mL penicillin and 100 mg/mL streptomycin. For MCF 10A, DMEN-F12 medium was used, supplemented with 10% fetal bovine serum, 100 µg/mL EGF, 1 mg/mL hydrocortisone, 10 mg/mL insulin, 100 U/mL penicillin and 100 mg/mL streptomycin. PC3, A549, HeLa and HUVEC cells were cultivated in RPMI 1640 medium supplemented with 10% fetal bovine serum, 2 mM L-glutamine, 2 mM sodium pyruvate, 1 mM nonessential amino acids, 100 U/mL penicillin and 100 mg/mL streptomycin, and maintained at 37 °C in 5% CO_2_. All the cell lines were confirmed as mycoplasma free by mycoplasma PCR tests.

### Animals

Balb-c mice (male, 6 weeks old and approximately 250gr) were supplied by the Federal University of Uberlandia and the procedures were approved by the Committee for the Ethical Use of Animals (CEUA) of the Federal University of Uberlandia (UFU), protocol number 042/14. All experiments were performed according to guidelines and regulations of Committee for the Ethical Use of Animals. None animal were excluded from the experimental groups.

### Cytotoxicity by MTT assay

The cells MDA-MB-231, MCF-7, PC3, A549, HeLa, MCF 10A and HUVEC, were seeded at 3 × 10^4^ cells per well in 96-well microplates. After 24 h a new medium containing γCdcPLI (50, 25, 12.5, 6.25, 3.125 or 1.560 µg/mL) or medium (control) was added and incubated at 37 °C and 5% CO_2_ for 24 h. After treatment the cells were incubated with MTT (5 mg/mL, 20 µL/well (MTT: 3-(4,5-dimethylthiazol-2-yl)-2,5-diphenyl tetrazolium bromide) for 3 h at 37 °C. Then, was added 100 µL/well of PBS containing 10% SDS and 0.01 M HCl (18 h, 37 °C and 5% CO_2_). The absorbance was read on a multi-well scanning spectrophotometer (Multiskan™ GO Microplate Spectrophotometer–Thermo Scientific, USA) at 570 nm. The IC_50_ value, which represents the concentration of protein that decreases viability to 50% (IC_50_), was calculated from the concentration-response curve.

### Apoptosis assay MDA-MB-231

Apoptosis was assessed by an apoptosis kit (BD Biosciences), according to the manufacturer’s instructions. Briefly, MDA-MB-231 cells were seeded at 2 × 10^6^ cells/well in 24-well plates and incubated with γCdcPLI (50 and 25 µg/mL) or medium (control) for 24 hours at 37 °C and 5% CO_2_. After incubation, cells were resuspended in binding buffer, stained with FITC-conjugated Annexin V/Propidium Iodide (PI) (2 μL from each FITC and PE-conjugated) and incubated in the dark for 15 minutes. Analyses were performed by the software BD Accuri C6 (BD Accuri C6–Biosciences, CA, USA).

### Gene expression analysis for Real-Time PCR

MDA-MB-231 and HUVEC cells were incubated with γCdcPLI at 25 µg/mL or medium (control group) for 24 hours (MDA-MB-231) and 1 hour (HUVEC) at 37 °C and 5% CO_2_. After incubation, the RNA was extracted from the adhering cells using the kit Tri-Reagent Sigma^®^ and stored at −80 °C. The RNA concentration and purity were estimated by optical density at 230 and 260 nm, respectively. For cDNA construction, the kit GoScript Reverse Transcription System (Promega^®^) was used according to the manufacturer’s instructions.

Gene expression was analyzed by quantitative real-time PCR (qRT-PCR). The analyses were made through the Human Cancer Pathway Primer Library, following the manufacturer’s instructions (GO-GenOne; Brazil; http://www.genone.com.br). Quantitative PCRs were carried out in triplicate using Master Mix–Sybr Green (LGC Biotechnology; Brazil), cDNA (2 μL) and 0.1 µM of each primer. The following thermal cycling protocol was used: 50 cycles at 95 °C (10 s) and 58 °C (45s). The data obtained were analyzed using comparative threshold cycle (CT) method according to Livak and Schmittgen^85^. Data were normalized using β-actin as a housekeeping gene; the results were expressed in fold changes (expression in γCdcPLI-treated groups compared to medium-treated groups).

### Western blotting

MDA-MB-231 cells were seeded in 6-well plates (10^6^cells/well) in triplicate and incubated overnight. Next, γCdcPLI (25 μg/mL) was incubated with cells for 24 h. After treatment, cells were washed with cold PBS, harvested in RIPA lysis buffer with protease inhibitor cocktail (sc-24948), vortexed extensively and centrifuged at 12,000 × g for 10 minutes at 4 °C. The amount of protein was verified by the method of Bradford^86^. Supernatant aliquots of proteins were resolved by sodium dodecyl sulfate-polyacrylamide gel electrophoresis (SDS-PAGE; 10% acrylamide/bis-acrylamide) and transferred onto nitrocellulose membranes (Hybond C, Amersham Biosciences). Membranes were blocked for 1 hour in TBS-T buffer (150 mM sodium chloride, 50 mM Tris [pH 8], and 0.1% Tween 20) containing 5% nonfat milk and were then incubated with antibodies recognizing phospho-Akt Ser473 (#4060), phospho-ERK1/2 Thr202/Tyr204 (#9101), ERK1/2 (#9102), phospho-p38 Thr180/Tyr182 (#4511), p38 (#9212), phospho-p53 Ser15 (#9284), p53 (#2524) and phospho-IKKα/IKKβ Ser176/Ser177 (#2078S) from Cell Signaling; Akt 1/2/3 (sc-8312), HPRT (sc-20975), GAPDH (sc-47724), Caspase 3 (sc-7148) and Caspase 7 (sc-56063) from Santa Cruz Biotechnology; phospho-IκBα Ser32/Ser36 (NB-100-56724) and IκBα (NB-120-22071) from Imgenex (San Diego, California, USA). After washing, membranes were incubated with anti-IgG secondary anti-rabbit (#474 1506) or anti-mouse (#04 18 06) antibodies conjugated to horseradish peroxidase from KPL; immunoreactive signals were visualized with enhanced chemiluminescence using SuperSignal chemiluminescent substrate (Life Technologies). All images were obtained using Uvitec Alliance documentation system.

### Cell adhesion inhibition assay

MDA-MB-231 and HUVEC cells (3 × 10^4^cells/well) were pre-incubated with different concentrations of γCdcPLI (50, 25, 12.5, 10, 6.25, 5, 3.125 and/or 2.5 µg/mL) or medium (control group) for 30 min at 37 °C. After incubation, cells were seeded into 96-well plates and incubated for 2 h at 37 °C in 5% CO_2_. Unattached cells were removed by washing with PBS, whereas the attached cells were measured by MTT assay, as described in item 2.4.

### Inhibition of HUVEC Adhesion to Extracellular Matrix Proteins

To evaluate the interaction between γCdcPLI and extracellular matrix components (ECM), collagen IV (10 μg/mL in 0.1 M of acetic acid), fibronectin (10 μg/mL in PBS), or matrigel (1 mg/mL in PBS) were coated on 96-well plates overnight at 4 °C and blocked with 1% of BSA. Afterwards, HUVEC cells (3 × 10^4^cells/well) pre-incubated with different concentrations of γCdcPLI (50, 25, 12.5, 10, 6.25, 5, 3.125 and/or 2.5 µg/mL) or medium (control group) for 30 min at 37 °C, cells were seeded and incubated for 2 h at 37 °C in 5% CO_2_. Unattached cells were removed by washing with PBS. Attached cells were quantified by the MTT assay as described previously.

### Wound healing assay

The inhibition of MDA-MB-231 migration was measured by a wound healing assay as described by Jung (2013) with modifications^87^. Briefly, cells were seeded at 4 × 10^6^ cells/well in 12-well plates. After 24 hours, the medium was discarded and the confluent monolayer was scratched with a 10 µL pipette tip to create an area devoid of cells. After this process, cells were treated with PLA_2_ inhibitor (γCdcPLI) at 25 µg/mL or medium (control group) for 24 hours and the confluence of cells was analyzed in an inverted optical microscope (Nikon Eclipse TS100).

### Transwell migration and invasion cell

The migration and invasion assays were performed using an 8μm pore cell culture insert (Greiner Bio-One, Switzerland). The HUVEC and MDA-MB-231 cells were pre-incubated in medium without FBS with γCdcPLI (50 and 25 µg/mL) for 30 min at 37 °C. After incubation, cells at 1 × 10^5^cell/transwell were seeded in upper chamber of the inserts. The lower chamber was filled with medium composed of 10% FBS. For the invasion assay, Matrigel (BD, USA) diluted at a 1:10 ratio (PBS:Matrigel, v/v) was coated on top of the chamber, 30 min before the seeding of the cells. The cells were maintained at 37 °C in a humidified incubator containing 5% CO_2_ for 24 hrs. Moreover, the non-migrating or invading cells were removed using a cotton swab. The migrated or invaded cells were stained with Panotic Kit (Laborclin, Brazil), the cells were photographed (Nikon Eclipse TS100) and counted. The positive control is represented by cells with medium and 10% FBS; the negative control is represented by cells with medium without FBS.

### Integrin quantification

HUVEC cells were pre-incubated with γCdcPLI (50 μg/mL) or medium (control group) for 1 hour at 37 °C in a humidified incubator containing 5% CO_2_. The cells were then blocked with BSA 0.5%, for 20 min on ice, washed and resuspended in PBS. Afterwards, the cells were incubated with anti-human α2 (CD49b) fluorescein, anti-human β1 (CD29) fluorescein, anti-human αVβ3 (CD51/CD61) fluorescein and anti-human α5 (CD49e) fluorescein monoclonal antibodies (Ambriex, Brazil) at a concentration of 2 µg/10^6^ cells in PBS with 1% BSA for 30 min/4 °C. All the cell suspensions were also incubated with the respective fluorescein-labeled Isotype control monoclonal antibodies. The samples were washed, resuspended in PBS and analyzed by the software BD Accuri C6 (BD Accuri C6–Biosciences, CA, USA).

### *In vitro* angiogenesis (HUVEC tube formation assay)

The vessel formation in HUVEC cells was evaluated by the Matrigel tube formation assay. The HUVEC cells (5 × 10^5^ cells/well) were pre-incubated with 25 µg/mL of γCdcPLI or medium (control group) for 30 min at 37 °C in RPMI medium supplemented with bFGF (10 ng/mL). Next, the cells were seeded on a cell culture chamber slide coated with 50 µl of Matrigel 5.25 mg/mL (Corning® Matrigel® Matrix, USA) and maintained at 37 °C in a humidified incubator containing 5% CO_2._ After 18 h cells were photographed in an inverted optical microscope (Nikon Eclipse TS100) and the vessels enumerated.

### Mouse aortic ring assay

This assay was performed according to Baker, *et al*., 2012 with modifications^74^. Aortic fragments (1 mm–1.5 mm) were removed from male Balb-c mice (eight animals per experiment, with 6 weeks old, according to statement approved by the Committee for the Ethical Use of Animals (CEUA) of the Federal University of Uberlandia (UFU), protocol number 042/14), and rinsed in ice-cold PBS supplemented with 1% penicillin-streptomycin. The fragments were placed on top of Matrigel (5.25 mg/mL, Corning® Matrigel® Matrix, USA) coated on 48-well plates and incubated in RPMI medium supplemented with bFGF (10 ng/mL) and EGF (20 ng/mL) for 24 h before being treated with one of several concentrations of γCdcPLI (50 and 25 μg/mL) or medium (control group). These fragments were treated over 7 days, at 2-day intervals. Subsequently, the aortic rings were photographed using an inverted optical microscope (Nikon Eclipse TS100).

### VEGF quantification

The levels of VEGF were quantified in HUVEC supernatants that were obtained from the *in vitro* angiogenesis assay and evaluated using a commercial CBA kit to solubilize proteins (BD–USA), according to the manufacturer’s protocol. The samples were analyzed by the software BD Accuri C6 (BD Accuri C6–Biosciences, CA, USA).

### Prostaglandin and Leukotriene assays (EIA assay)

Prostaglandin (PGE2) and leukotriene (LTB4) levels were evaluated in culture supernatants using a commercial enzyme immunoassay kit (EIA Kit–Cayman; USA), according to the manufacturer’s protocol. To obtain the supernatants, MDA-MB-231 cells were seeded at 4 × 10^6^cells/well in 12-well plates; after 24 hours the medium was discarded and a new medium with γCdcPLI (25 µg/mL) or IMDM medium (control group) was added for 24 hours and collected for PGE2 and LTB4 quantification.

### Statistics

Experiments were carried out in triplicate, and the results were expressed as mean ± S.E.M. Differences between treatments and controls were analyzed by the Student’s *t*-test (Unpaired or Nonparametric test, assuming normal Gaussian distributions) or One-Way ANOVA, whereas the comparison of two or more variables was assessed by the Two-Way ANOVA; when possible all tests were followed by the Bonferroni post-test, using the software GraphPad Prism (GraphPad Software, Inc., San Diego, USA). Differences between groups were considered statistically significant at **p < 0.05 and ***p < 0.001. In addition, the variance was similar with the groups and statistically compared, each experimental replicate were designed randomly and when possible the investigator stay blinded to the group during the results analyses.

### Data availability

All relevant data supporting the findings of this study are available within the article, in Supplementary Information, or from the authors on request.

This research was registered in IBAMA (Instituto Brasileiro do Meio Ambiente e dos Recursos Naturais Renováveis, Brazil) under the number 010632/2014-0.

The newly reported inhibitor and its use are claimed under the patent application BR1020160309468, filed on December 29th, 2016, with authorship of SCG; DSL; FVPVA; MAS; LRG; ERV; PTA; TCSR; VLCB; ALQS; RSR; KAGY; VMRA

## Electronic supplementary material

Supplementary
