# Supplementary material for: Antitumoral effects of γCdcPLI, a PLA2 inhibitor from Crotalus durissus collilineatus via PI3K/Akt pathway on MDA-MB-231 breast cancer cell
Source: Sci Rep. 2017 Aug 1;7:7077. doi: 10.1038/s41598-017-07082-2 (PMC5539153; doi:10.1038/s41598-017-07082-2)
Supplement: Supplementary file 1 — Supplementary [file 41598_2017_7082_MOESM1_ESM.docx]

Antitumoral effects of γCdcPLI, a PLA_2_ inhibitor from *Crotalus durissus collilineatus* via PI3K/Akt pathway on MDA-MB-231 breast cancer cell

Sarah N. C. Gimenes^1^; Daiana S. Lopes^1^; Patrícia T. Alves^1^; Fernanda V. P. V. Azevedo^1^; Lara Vecchi^1^; Luiz R. Goulart^1^; Thais C. S. Rodrigues^1^; André L. Q. Santos^1^, Vera L. de C. Brites^1^; Thaise L. Teixeira^1^; Cláudio V. da Silva^1^; Matheus H. Dias^2^; Samuel C. Teixeira^1^; Renata S. Rodrigues^1^; Kelly A. G. Yoneyama^1^; Ricardo A. Oliveira^1^; Veridiana de M. Rodrigues^1^*

^1^Federal University of Uberlandia, Uberlandia, MG, Brazil. ^2^ Butantan Institute, São Paulo, Brazil

**Supplementary Information**

**Supplementary Figure 1**

**
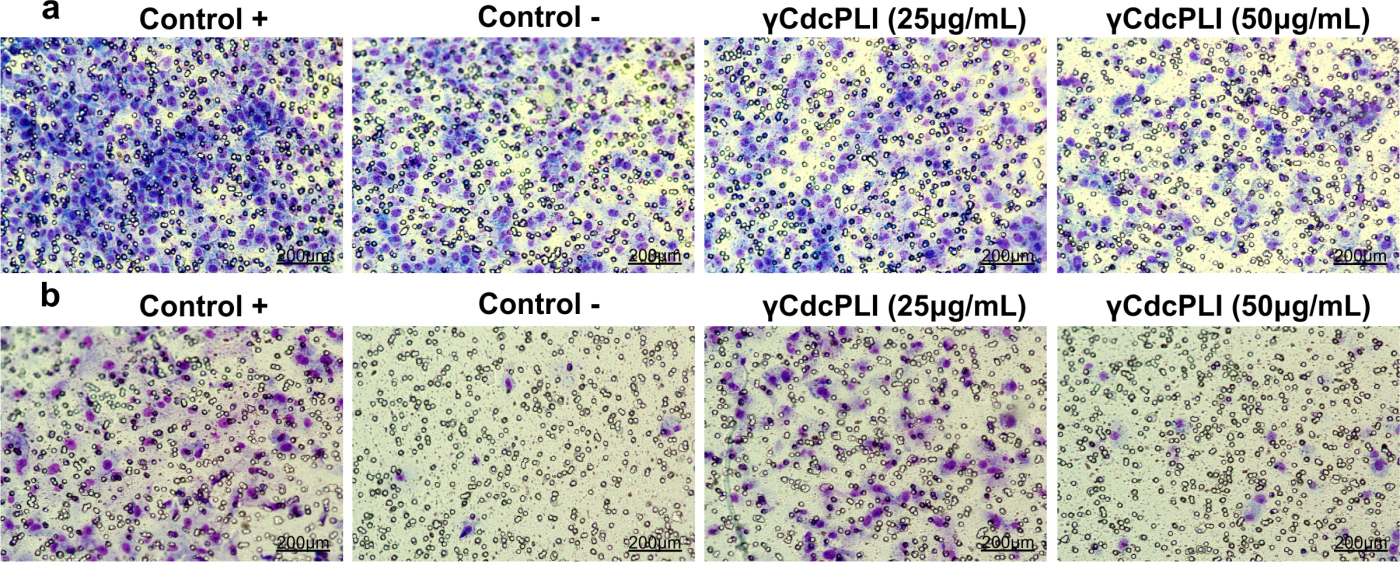
**

**Supplementary Figure 1:** **Representative migration and invasion analysis by Transwell assay in MDA-MB-231 cells.** The positive control was represented by cells with SFB 10%, negative control represented by cells without SFB and the cells treated γCdcPLI at 25 and 50μg/mL. The migrated or invaded cells were stained with Panotic Kit and photographed using Nikon Eclipse TS100 / 20X and counted.

**Supplementary Figure 2**





**Supplementary Figure 2: Representative migration analysis by Transwell assay in HUVEC cells.** The positive control was represented by cells with SFB 10%, negative control represented by cells without SFB and the cells treated γCdcPLI at 25 and 50μg/mL. The migrated cells were stained with Panotic Kit and the cells were photographed using Nikon Eclipse TS100 / 20X and counted.

**Supplementary Figure 3**


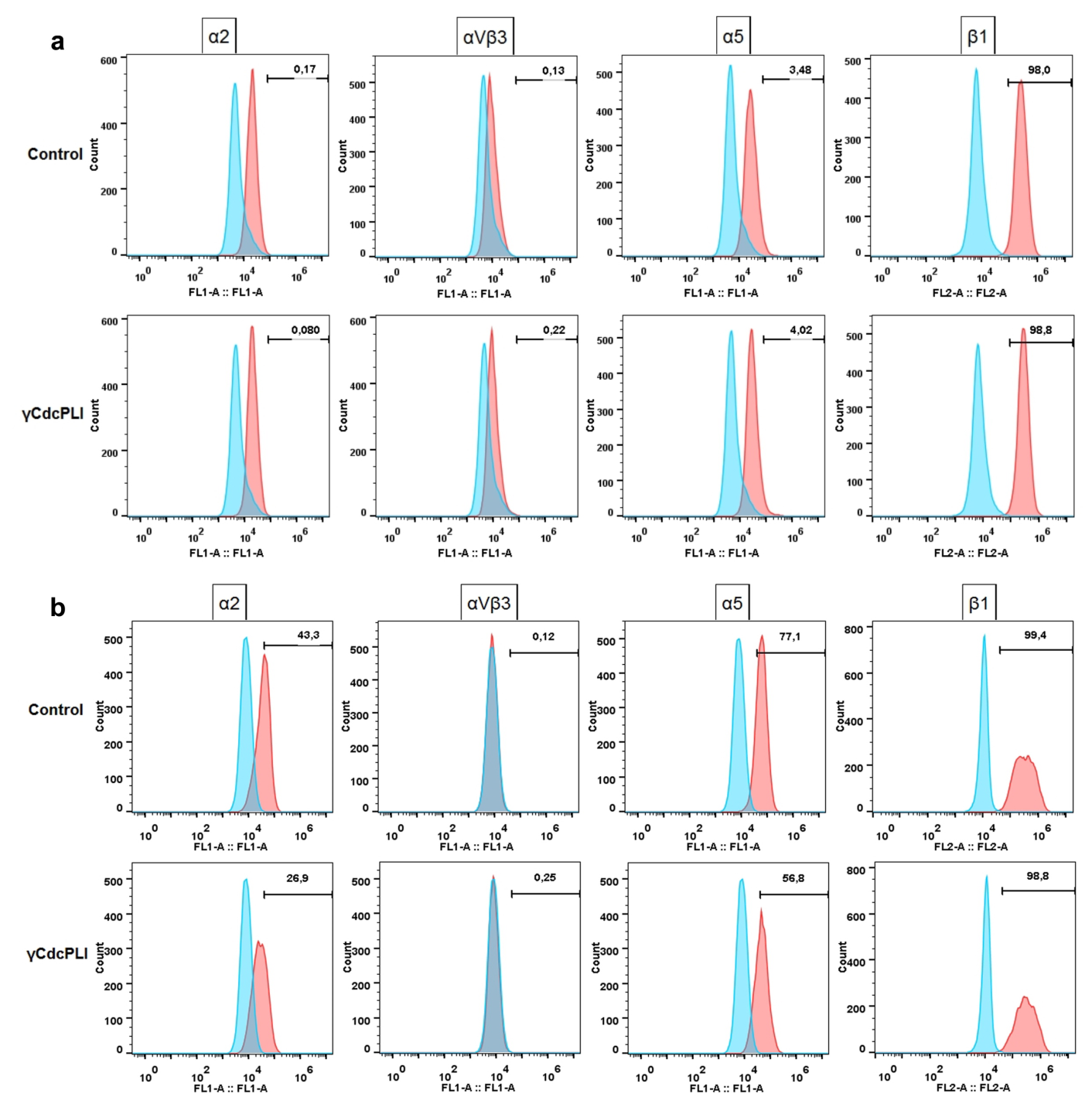


**Supplementary Figure 3: The representative graphical from FlowJo program profile of integrin expression**. **a)** Integrin expression in MDA-MB-231 cells and, **b)** Integrin expression in HUVEC cells. The samples were analyzed by the software BD Accuri C6.
